# Diet quality and Parkinson’s disease: Potential strategies for non-motor symptom management

**DOI:** 10.1016/j.parkreldis.2023.105816

**Published:** 2023-08-19

**Authors:** Dayoon Kwon, Aline D. Folle, Irish Del Rosario, Keren Zhang, Kimberly C. Paul, Adrienne M. Keener, Jeff M. Bronstein, Beate Ritz

**Affiliations:** a Department of Epidemiology, UCLA Fielding School of Public Health, Los Angeles, CA, USA; b Department of Neurology, UCLA David Geffen School of Medicine, Los Angeles, CA, USA

**Keywords:** Parkinson’s disease, Diet quality, Nutrients, Phenotype, Constipation

## Abstract

**Introduction:**

Parkinson’s disease (PD) is now considered a systemic disease, and some phenotypes may be modifiable by diet. We will compare the diet quality and intake of specific nutrients and food groups of PD patients with household and community controls to examine how diet may influence PD clinical features.

**Methods:**

We conducted a case-control study of 98 PD patients and 83 controls (household = 53; community = 30) in central California, assessing dietary habits over the past month and calculating the Healthy Eating Index (HEI)-2015. We employed multivariate logistic and linear regression analyses to assess associations between diet and PD status, PD symptom profiles, and medication, adjusting for relevant confounders.

**Results:**

PD patients had a lower HEI score than controls, with an OR of 0.65 (95% CI: 0.45, 0.94) per 10-points increase in HEI. Lower-quality diet was characterized by higher intakes of carbohydrates, total and added sugars, and trans fats and lower intakes of fiber, folate, unsaturated fatty acids, protein, and fat. PD patients with chronic constipation had a 4.84 point lower HEI score than those without (β per 10-point in HEI: −0.48; 95% CI: −0.97, −0.00). Furthermore, patients on high dopamine agonist doses consumed more sugar than those on lower doses.

**Conclusion:**

PD patients consume a lower-quality diet compared to household and community controls. Dietary modifications may alleviate non-motor symptoms like constipation, and promoting a healthy diet should become a part of routine care and disease management for PD patients, with special attention on agonist-treated and hyposmic patients.

## 1. Introduction

In Parkinson’s Disease (PD), a complex neurodegenerative disease with increasing incidence and prevalence [1], patients suffer from progressive motor and non-motor features, including constipation and hyposmia. Non-motor symptoms are recognized as significant aspects of PD that substantially impact the overall quality of life. With an aging global population and limited treatment options, there is a growing need to promote healthy aging and develop personalized management strategies for PD patients.

Diet plays a critical role in managing PD, e.g., a fiber-rich diet for constipation. PD patients are also advised to follow a low-protein diet to prevent dietary amino acids from competing with levodopa absorption and reducing medication effectiveness [2]. Patients may modify their eating behavior and lose appetite due to the loss of smell and taste [3]. Some medications, particularly dopamine agonists, may influence eating behaviors through weakened impulse control [4]. Thus, the interplay between non-motor symptoms, such as smell loss, and medication-associated effects complicates our understanding of eating habits in PD patients.

Research suggested that dietary patterns represented by the Alternate Healthy Eating Index (AHEI) or alternate Mediterranean Diet score (aMED) may benefit PD patients, reduce PD risk [5] and possibly alleviate non-motor symptoms [6]. It has been reported that PD patients consume high amounts of sugars and carbohydrates [7], with potential impacts on quality of life and symptom severity.

Previous prospective studies examined the diet of PD patients among healthcare professionals [5,6], a special subgroup unlikely to represent the larger PD community. Our objective is to describe differences in diet quality, specific nutrients, and foods in community-based PD patients compared to household and community controls in central California. We also investigate dietary differences with non-motor symptoms and PD medications to potentially identify dietary strategies for improving symptom management and slowing disease progression.

## 2. Methods

### 2.1. Study population

The Parkinson’s Environmental and Gene (PEG) study is a population-based case-control study of PD conducted in Kern, Tulare, and Fresno counties, California. Eligible PD cases were newly diagnosed (within 3–5 years), residents of California for at least five years, with diagnostic confirmation by the University of California at Los Angeles (UCLA) movement disorder specialists, without other neurological conditions or terminal illnesses, and who provided consent to participate. Community controls were randomly selected according to residential addresses in the same counties using tax assessor and Medicare lists.

Since 2017, we have conducted a sub-study focusing on the microbiome (PEG-GUT), and the study’s findings will soon be available for publication. Participants were re-contacted to collect fecal samples and dietary information. Controls were recruited: household members of PD cases to account for shared environments and community members to avoid excessive matching on environmental and lifestyle factors within households. PEG-GUT included 125 confirmed PD cases and 97 controls (64 household and 33 community members). We excluded participants with missing food frequency questionnaires (n = 39) and those with implausible daily energy intakes (men: <500 or >5000 kcal/d, women: <400 or >4000 kcal/d; n = 2). Cross-sectional analyses were based on 98 cases and 83 controls (53 household and 30 community members). The excluded individuals (27 cases and 14 controls) were observably similar to the 181 included in the analyses. This study was approved by UCLA Human Subjects Committee.

### 2.2. Assessments

Participants’ age, gender, education, weight, height, and smoking status were collected via interviews. Body mass index (BMI) was calculated as weight (kg)/height squared (m^2^). Dietary intake for the past month was assessed using the National Cancer Institute Diet History Questionnaire (DHQ) II (134 food items and 8 dietary supplement questions). Participants reported their average frequency of consumption over the past month (‘never’ to ‘six or more times per day’), and these responses were converted to daily intake. Primary analyses used the Healthy Eating Index (HEI)-2015; secondary analyses the AHEI-2010 and aMED (Table S1). Additionally, we assessed dietary components including 1) **nutrients**: carbohydrates, total protein, total fat, trans fats, monounsaturated fatty acids (MUFA), polyunsaturated fatty acids (PUFA), total sugars, added sugars, fiber, folate, alcohol, and caffeine; and 2) **food groups**: vegetables, fruit, refined grains, nuts, and processed meat.

Constipation status was measured using the Wexner Constipation Scoring System (no: ≤5 points; yes: >5 points). PD patients self-reported chronic disorders, including chronic constipation that required laxative use; non-motor symptoms with the Non-Motor Symptom Assessment Scale and the Geriatric Depression Scale-15. PD medications were evaluated as levodopa and dopamine agonists and used to calculate the levodopa equivalent daily dose (LED). Motor symptom severity was based on the MDS-Unified Parkinson’s Disease Rating Scale (MDS-UPDRS) part III. Patients reported non-motor and motor experiences of daily living (mentation, behavior, and mood) according to the MDS-UPDRS parts IA, IB, and II. Global cognitive function was evaluated with the Mini-Mental State Examination, a universally accepted test for detecting cognitive dysfunction, but with limitations like ceiling effects and inadequate sensitivity in detecting executive function problems.

### 2.3. Statistical analysis

First, we examined the associations of dietary patterns and components with PD status using unconditional logistic regression. Matches were broken to include a larger number of cases and controls. Dietary patterns and components were modeled as continuous variables per unit increase (e.g., 1, 10, 100). We controlled for age (years), gender, smoking status (never, ever), BMI (kg/m^2^), and total energy intake (kcal/d).

Second, using linear regression models, we evaluated diet differences and the influence of PD duration and age at diagnosis on diet. We compared dietary habits between PD patients with and without non-motor symptoms, as well as those on high and low dose medication. All models were adjusted for age and gender. In sensitivity analyses, we stratified by constipation status and gender, removed household controls, or used conditional logistic regression to account for county (community) and household pairs matching. All analyses were performed using R version 4.2.2.

## 3. Results

### 3.1. Demographic and clinical characteristics

Participants’ average age at diet assessment was 73 years, with 67% being men with PD and 64% being women as controls, reflecting the higher incidence of PD in men and mostly spouses serving as household controls (Table 1). Most participants were of European descent, well-educated, never-smokers, and slightly overweight. PD patients had less healthy diets than controls and a high proportion suffered from constipation (52%). The mean age at PD diagnosis was 64 years, and the mean PD duration at the interview was nine years. Most patients were taking levodopa (88%) and/or dopamine agonist (68%) medications, with a majority exhibiting the postural instability and gait impairment PD phenotype (64%) (Table S2).

### 3.2. Dietary characteristics in PD patients versus controls

**Fig. 1.**
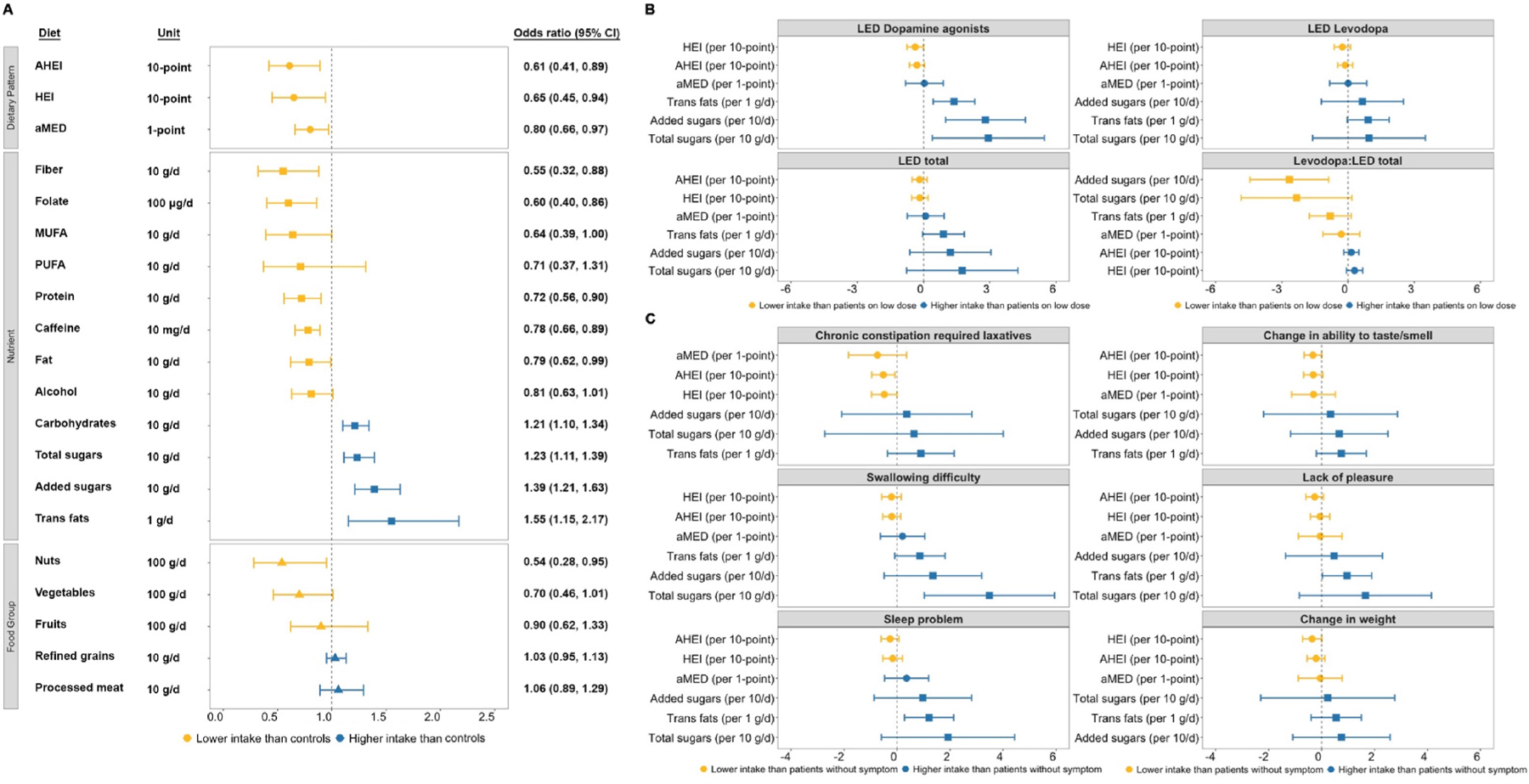
Diet and Parkinson’s disease. (**A**) Associations of dietary patterns and components with Parkinson’s disease status (N = 181). Coefficients are odds ratios estimated from logistic regressions. Adjusted for age, gender, smoking, BMI, and energy intake. (**B**) Associations of Parkinson’s disease medications (high vs. low dose) with dietary patterns and specific nutrients (n = 98). Coefficients are from linear regressions of medications on dietary patterns and specific nutrients. Adjusted for age and gender. LED dichotomized into high and low doses for dopamine agonists (high: ≥200 mg; low: <200), levodopa (high: ≥500 mg; low: <500), total (high: ≥600 mg; low: <600), and Levodopa:LED total (high: ≥0.8; low: <0.8). (**C**) Associations of Parkinson’s disease non-motor symptoms (present vs. absent) with dietary patterns and specific nutrients (n = 98). Coefficients are from linear regressions of non-motor symptoms on dietary patterns and specific nutrients. Adjusted for age and gender. Parkinson patients self-reported chronic constipation requiring laxative use. Other non-motor symptoms assessed using the Non-Motor Symptom Assessment Scale. *Abbreviations*: **CI**, confidence interval; **AHEI**, Alternate Healthy Eating Index; **HEI**, Healthy Eating Index; **aMED**, alternate Mediterranean Diet score; **MUFA**, monounsaturated fatty acids; **PUFA**, polyunsaturated fatty acids.

In multivariable logistic regression analyses, a lower score for the dietary indices was associated with PD; for example, we estimated an OR of 0.65 (95% CI: 0.45, 0.94; Fig. 1A) per 10-point increase in the HEI. Regarding nutrient intakes, PD patients, compared to controls, consumed more carbohydrates, total sugars, added sugars, and trans fats; but less fiber, folate, unsaturated fatty acids, protein, and fat. As expected, PD patients reported lower intakes of alcohol and caffeine than controls. Additionally, in terms of food groups, PD patients consumed lower amounts of nuts compared to controls.

Stratifying by constipation status, positive associations with sugars and carbohydrates and negative associations with protein and folate remained in constipated PD patients (Table S3). PD patients consumed more total sugars compared to controls, with stronger associations observed in the constipated group and among men (Table S4). Using conditional logistic regression for matched pairs did not materially change the magnitude of associations between dietary patterns and PD (Table S5), nor did excluding household controls.

### 3.3. Dietary intake among PD patients only

In multivariable linear regression analyses, PD patients younger at diagnosis or with longer disease duration tended to consume greater amounts of total sugars, added sugars, and trans fats (Table S6). Higher doses of dopamine agonists were associated with increased sugar and trans fat consumption, while higher levodopa to total LED ratio was associated with lower added sugar intake (Fig. 1B). Patients on higher doses of dopamine agonists had a 28.04 g/d higher intake of added sugars compared to those on lower doses. Patients with swallowing difficulties also reported higher total sugar intake, while those with a lack of pleasure and sleep problems consumed more trans fats.

Lower HEI scores were associated with non-motor symptoms, such as chronic constipation and hyposmia, in PD patients (Fig. 1C), i.e., a 4.84-point lower HEI score in patients with chronic constipation compared to those without (β per 10-point in HEI: −0.48, 95% CI: −0.97, −0.00). PD patients with cognitive impairment had lower protein, fiber, and folate intake than those without (Table S7).

## 4. Discussion

Studying dietary habits of PD patients with an average disease duration of nine years, we found that they had less healthy diets compared to their household members and community controls. They consumed more carbohydrates, total sugars, added sugars, and trans fats; but less fiber, folate, unsaturated fatty acids, protein, and fat. Longer disease duration and high dopamine agonist doses were associated with increased sugars and trans fats; non-motor symptoms, such as chronic constipation and hyposmia, with lower healthy diet scores. These findings suggest that diet quality may deteriorate with PD duration and progression, potentially influenced by non-motor symptoms and medication use.

Our findings align with previous investigations in large US cohorts, linking a low-quality diet to PD risk [5] and constipation in PD [6]. As fiber promotes regular bowel movements, PD patients need to be advised to include fiber-rich foods in their diet. Also, PD patients with hyposmia should be especially encouraged to maintain their overall dietary quality.

The specific mechanism of how a low-quality diet affects PD or its features during progression is still unclear, but emerging evidence suggests an involvement of the brain-gut axis [8]. Poor adherence to a healthy diet may contribute to α-synuclein aggregation in the gut, disruption of the enteric nervous system, and a lack of antioxidants and anti-inflammatory compounds.

Altered eating behaviors in PD may be due to changes in hypothalamic regulation, energy homeostasis, and dopaminergic signaling. Similar to us, an Australian study found that PD patients consumed more added sugar [7]. This could be a compensatory mechanism for disease-related dopamine loss, as sugar increases brain dopamine through insulin and alters brain activity in reward-processing regions [9]. Higher sugar consumption was also associated with a higher dopamine agonist dosage, linked to impulse control disorders and increased sugar cravings, affecting impulse control and decision-making processes related to food choices [4]. Our PD patients tended to consume less caffeine and alcohol, possibly replacing them with juice or sugar-sweetened drinks [10].

In an Italian PD case-control study, swallowing difficulties were not associated with a healthy diet but with softer and more viscous food, including sweetened yogurt, milk puddings, and custards [11]. Our study found that PD patients with swallowing difficulty consumed higher amounts of total sugars, consistent with Italian sweetened and sugary foods.

Our PD patients consumed more trans fats than controls, which are known contributors to type 2 diabetes mellitus (T2DM) but not PD. In healthy mice fed a high-fat diet, the induction of peripheral insulin resistance showed nigrostriatal dopaminergic dysfunction and parkinsonism, suggesting a connection between a high-fat diet, insulin resistance, and PD [12]. These findings support a connection between PD and T2DM, as both conditions share dysregulated pathophysiological pathways.

While our study’s cross-sectional design limits determining directionality for observed associations, many findings are supported by prospective or case-control diet studies in PD. The DHQ may have introduced misclassification due to recall errors for food types or portion sizes. Comprehensive clinical assessments by movement disorder specialists ensured accurate PD diagnoses. Our study population was primarily white non-Hispanic, limiting generalizability to other racial/ethnic groups.

In summary, our study suggests that PD patients in central California counties have lower dietary quality compared to their household and community controls. Lower adherence to a healthy diet was associated with non-motor features of PD, particularly chronic constipation and hyposmia. PD patients on dopamine agonist treatment tended to consume excess amounts of total sugars, added sugars, and trans fats. Diet recommendations that encourage healthy choices within routine patient care may help avoid some PD complications.

## Supplementary Material

Supplemental Material

## Figures and Tables

**Table 1:** Characteristics of Parkinson’s disease cases and controls (N = 181).

Characteristic	Cases	Controls
	(n = 98)	(n = 83)
**Age**, years		
Mean (SD)	73.7 (9.1)	71.3 (8.2)
**Gender**		
Men	66 (67.3%)	30 (36.1%)
Women	32 (32.7%)	53 (63.9%)
**Race**		
White	82 (83.7%)	68 (81.9%)
Non-White	16 (16.3%)	15 (18.1%)
**Body mass index**, kg/m^2^		
Normal, <25	35 (35.7%)	27 (32.5%)
Overweight, 25–29.9	37 (37.8%)	35 (42.2%)
Obesity, ≥30	26 (26.5%)	21 (25.3%)
**Education**, years		
Mean (SD)	15.6 (3.8)	15.8 (3.0)
**Smoking status**		
Never	62 (63.3%)	60 (72.3%)
Ever	36 (36.7%)	23 (27.7%)
**Constipation**		
No	47 (48.0%)	58 (69.9%)
Yes	51 (52.0%)	25 (30.1%)
**Energy intake**, kcal/d		
Mean (SD)	1970.2 (889.1)	1737.9 (790.1)
**HEI-2015**		
Mean (SD)	63.5 (8.9)	66.7 (9.2)
**AHEI-2010**		
Mean (SD)	70.3 (8.1)	74.3 (9.4)
**aMED**		
Mean (SD)	3.9 (2.0)	4.2 (1.9)

*Abbreviations*: **SD**, standard deviation; **HEI**, Healthy Eating Index; **AHEI**, Alternate Healthy Eating Index; **aMED**, alternate Mediterranean Diet score.
