# Supplementary material for: Diet quality and Parkinson’s disease: Potential strategies for non-motor symptom management
Source: Parkinsonism Relat Disord. Author manuscript; Available in PMC 2024 May 24. (PMC11121503; doi:10.1016/j.parkreldis.2023.105816)
Supplement: Supplemental Material [file NIHMS1990725-supplement-Supplemental_Material.pdf]

Supplementary materials

Diet quality and Parkinson's disease: potential strategies for non-motor symptom management

D Kwon et al.

Supplementary materials are divided into two sections:

Section I reports further details on dietary patterns.

Section II reports additional analyses supporting those reported in the main text of this article.

I. Dietary patterns

The National Cancer Institute questionnaire allowed us to generate three different diet quality scores to assess dietary patterns: the HEI-2015 [1], AHEI-2010 [2], and aMED [3] according to validated analytical procedures for this instrument (Table S1). Specifically, the HEI-2015 was developed based on the Update of the Healthy Eating Index [1] and contains 13 components (total vegetables, greens and beans, total fruit, whole fruit, whole grains, refined grains, dairy, total meat, seafood or plant protein, fatty acids, polyunsaturated to saturated fatty acid ratio, sodium, and added sugars) reflecting recommendations based on 2015-2020 Dietary Guidelines for Americans. The intake of foods and nutrients is represented on a density basis, as amount per 1,000 kcal. Each component is scored from 0 (indicating worst diet quality) to 5 or 10 (indicating best diet quality). Better scores are given to a higher intake of vegetables, fruits, whole grains, dairy, seafood or plant protein, polyunsaturated to saturated fatty acid ratio; a lower intake of refined grains, total meat, and added sugars. Thus, the total HEI ranges from 0 (worst) to 100 (best) points.

The AHEI was developed to improve on disease risk prediction [2]. The AHEI-2010 includes 11 components (vegetables, fruits, whole grains, juice, nuts and legumes, red and processed meat, trans fats, long-chain fats, polyunsaturated fatty acid, sodium, and alcohol), each with a score of 0 (indicating worst diet quality) to 10 (indicating best diet quality) and a total score ranging from 0 (worst) to 110 (best) points. Better scores are assigned to a higher intake of vegetables, fruits, whole grains, nuts and legumes, long-chain fats, and polyunsaturated fatty acids; a lower intake of juice, red and processed meat, trans fats, and sodium; and a light to moderate intake of alcohol.

The aMED is based on the traditional Mediterranean diet scale in the Greek population [4, 5]. This scale was modified using a food frequency questionnaire developed for the United States population's diet [3, 6] and is based on the intake of 9 components (vegetables without potato products, fruits, whole grains, nuts, legumes, fish, polyunsaturated to saturated fatty acid ratio, red and processed meat, and alcohol). For intake of the first seven items above the median of all study subjects, a person receives 1 point (indicating adherence); all others reporting less receive a 0 (indicating nonadherence). For alcohol, 1 point is assigned for intake between 5 and 15 g/d (Table S1). For red and processed meat, an intake below the median receives 1 point. Adherence

corresponds to a higher intake of vegetables, fruits, whole grains, nuts, legumes, fish, and polyunsaturated to saturated fatty acid ratio; a lower intake of red and processed meat; and a light to moderate intake of alcohol. The total score ranges from 0 (worst) to 9 (best) points.

Table S1. Criteria for scoring HEI-2015, AHEI-2010, and aMED scores

Food Group	HEI-2015 (0-100 points)		AHEI-2010 (0-110 points)		aMED (0-9 points)	
	Minimum	Maximum	Minimum	Maximum	Minimum	Maximum
Total vegetables	0 points<br>0 cups/1000 kcal	5 points<br>≥ 1.1 cups/1000 kcal	0 points<br>0 servings/day	10 points<br>≥ 5 servings/day	0 points<br>< median	1 point<br>≥ median
Greens & beans	0 points<br>0 cups/1000 kcal	5 points<br>≥ 0.2 cups/1000 kcal				
Total fruit	0 points<br>0 cups/1000 kcal	5 points<br>≥ 0.8 cups/1000 kcal	0 points<br>0 servings/day	10 points<br>≥ 4 servings/day	0 points<br>< median	1 point<br>≥ median
Whole fruit	0 points<br>0 cups/1000 kcal	5 points<br>≥ 0.4 cups/1000 kcal				
Whole grains	0 points<br>0 oz/1000 kcal	10 points<br>≥ 1.5 oz/1000 kcal	0 points<br>0 g/day	10 points<br>75 g/day (women)<br>90 g/day (men)	0 points<br>< median	1 point<br>≥ median
Refined grains	0 points<br>≥ 4.3 oz/1000 kcal	10 points<br>≤ 1.8 oz/1000 kcal				
All dairy	0 points<br>0 cups/1000 kcal	10 points<br>≥ 1.3 cups/1000 kcal				
Juice			0 points<br>≥ 1 servings/day	10 points<br>0 servings/day		
Total meat	0 points<br>0 oz/1000 kcal	5 points<br>≥ 2.5 oz/1000 kcal				
Nuts & legumes<br>(2 different categories for AMED)			0 points<br>0 servings/day	10 points<br>≥ 1 servings/day	0 points<br>< median<br>0 points<br>< median	1 point<br>≥ median<br>1 point<br>≥ median
Red/processed meat			0 points<br>≥ 1.5 servings/day	10 points<br>0 servings/day	0 points<br>≥ median	1 point<br>< median
Seafood or plant protein	0 points<br>0 oz/1000 kcal	5 points<br>≥ 0.8 oz/1000 kcal				
Fish					0 points<br>< median	1 point<br>≥ median
Trans fats			0 points<br>≥ 4% energy	10 points<br>≤ 0.5% energy		

Food Group	HEI-2015 (0-100 points)		AHEI-2010 (0-110 points)		aMED (0-9 points)	
	Minimum	Maximum	Minimum	Maximum	Minimum	Maximum
Long-chain fats			0 points<br>0 mg/day	10 points<br>250 mg/day		
PUFA			0 points<br>≤ 2% energy	10 points<br>≥ 0.5% energy		
MUFA:SFA					0 points<br>< median	1 point<br>≥ median
(MUFA+PUFA)/SFA	0 points<br>≤ 1.2	10 points<br>≥ 2.5				
Saturated fats	0 points<br>≥ 16% energy	10 points<br>≤ 8% energy				
Sodium	0 points<br>≥ 2.0 g/1000 kcal	10 points<br>≤ 1.1 g/1000 kcal	0 points<br>Highest decile	10 points<br>Lowest decile		
Alcohol			0 points	10 points	0 points<br><5 or >15 g/day	1 point<br>5-15 g/day
Women			≥ 2.5 drinks/day	0.5-1.5 drinks/day		
Men			≥ 3.5 drinks/day	0.5-2.0 drinks/day		
Added sugars	0 points<br>≥ 26% energy	10 points<br>≤ 6.5% energy				

Abbreviations: **HEI**, Healthy Eating Index; **AHEI**, Alternate Healthy Eating Index; **aMED**, alternate Mediterranean Diet score; **MUFA**, monounsaturated fatty acids; **SFA**, saturated fatty acids; **PUFA**, polyunsaturated fatty acids.

II. Supplementary analyses

Table S2. Parkinson's disease clinical characteristics (n=98)

Clinical characteristic	Cases (n=98)
**Age at diagnosis**, years	
Mean (SD)	63.6 (9.9)
Median (Min, Max)	63.5 (34.0, 84.0)
**PD duration**, years	
Mean (SD)	9.3 (4.8)
Median (Min, Max)	8.0 (0.0, 20.0)
**PD phenotype**	
Tremor Dominant	27 (27.6%)
Postural instability and gait impairment	63 (64.3%)
Indeterminate	8 (8.2%)
**MDS-UPDRS**	
**Part IA** (non-motor)	
Mean (SD)	3.6 (3.1)
Median (Min, Max)	3.0 (0.0, 14.0)
**Part IB** (non-motor)	
Mean (SD)	10.3 (4.9)
Median (Min, Max)	10.0 (1.0, 24.0)
**Part II** (motor)	
Mean (SD)	16.4 (8.3)
Median (Min, Max)	15.9 (1.0, 41.0)
**Part III** (motor)	
Mean (SD)	27.4 (12.5)
Median (Min, Max)	27.0 (3.0, 58.2)
**Non-Motor Symptom Assessment Scale**	
Mean (SD)	51.2 (44.2)
Median (Min, Max)	38.5 (0.0, 172.0)
**Mini Mental State Exam**	
Mean (SD)	27.9 (3.0)
Median (Min, Max)	29.0 (7.0, 30.0)
**Geriatric Depression Scale-15**	
Mean (SD)	3.8 (3.0)
Median (Min, Max)	3.0 (0.0, 12.0)
**Wexner score**	
Mean (SD)	6.8 (3.7)
Median (Min, Max)	6.0 (0.0, 16.0)
**Chronic constipation required laxatives**	
No	66 (67.3%)
Yes	32 (32.7%)

Clinical characteristic	**Cases** (n=98)
**Depression**	
No	64 (65.3%)
Yes	34 (34.7%)
**REM sleep behavior disorder**	
No	77 (78.6%)
Yes	21 (21.4%)
**Dopamine agonists use**	
No	31 (31.6%)
Yes	67 (68.4%)
**Levodopa use**	
No	12 (12.2%)
Yes	86 (87.8%)
**LED Dopamine agonists**, mg	
Mean (SD)	158.9 (169.7)
Median (Min, Max)	115.0 (0.0, 800.0)
**LED Levodopa**, mg	
Mean (SD)	638.1 (539.7)
Median (Min, Max)	510.6 (0.0, 2793.0)
**LED total**, mg	
Mean (SD)	800.1 (605.5)
Median (Min, Max)	680.6 (0.0, 3133.0)
**Levodopa:LED total**	
Mean (SD)	0.7 (0.3)
Median (Min, Max)	0.8 (0.0, 1.0)

Abbreviations: **PD**, Parkinson's disease; **SD**, standard deviation; **MDS, UPDRS**, Movement Disorder Society, Unified Parkinson's Disease Rating Scale; **LED**, Levodopa Equivalent Daily Dose.

Table S3. Association of dietary patterns and components with Parkinson's disease status according to constipation (N = 181)

		Constipation	
		**No**	**Yes**
**Cases/controls**		47/58	51/25
**Dietary pattern**	**Unit**	**OR (95% CI)[a]**	**OR (95% CI)[a]**
HEI	10-point	0.66 (0.39, 1.07)	0.76 (0.40, 1.44)
AHEI	10-point	0.60 (0.34, 1.01)	0.60 (0.30, 1.14)
aMED	1-point	0.85 (0.64, 1.11)	0.78 (0.54, 1.11)
**Nutrient**			
Carbohydrates	10 g/d	1.13 (1.02, 1.28)	1.45 (1.17, 1.89)
Protein	10 g/d	0.86 (0.62, 1.16)	0.40 (0.21, 0.65)
Fat	10 g/d	0.84 (0.63, 1.10)	0.72 (0.44, 1.13)
Trans fats	1 g/d	1.80 (1.12, 3.06)	1.31 (0.87, 2.11)
MUFA	10 g/d	0.73 (0.40, 1.26)	0.53 (0.21, 1.28)
PUFA	10 g/d	0.62 (0.27, 1.33)	1.17 (0.34, 4.19)
Total sugars	10 g/d	1.17 (1.04, 1.35)	1.35 (1.11, 1.74)
Added sugars	10 g/d	1.34 (1.13, 1.68)	1.39 (1.12, 1.81)
Fiber	10 g/d	0.57 (0.27, 1.08)	0.49 (0.21, 1.11)
Folate	100 μg/d	0.68 (0.39, 1.07)	0.54 (0.28, 0.95)
Alcohol	10 g/d	0.84 (0.62, 1.10)	0.69 (0.41, 1.15)
Caffeine	100 mg/d	0.80 (0.64, 0.97)	0.68 (0.49, 0.87)
**Food group**			
Vegetables	100 g/d	0.69 (0.36, 1.14)	0.65 (0.35, 1.17)
Fruits	100 g/d	0.86 (0.48, 1.52)	0.93 (0.54, 1.63)
Refined grains	10 g/d	1.02 (0.93, 1.15)	1.02 (0.87, 1.19)
Nuts	100 g/d	0.61 (0.26, 1.21)	0.57 (0.20, 1.70)
Processed meat	10 g/d	1.04 (0.83, 1.33)	1.03 (0.75, 1.45)

Abbreviations: **OR**, odds ratio; **CI**, confidence interval; **HEI**, Healthy Eating Index; **AHEI**, Alternate Healthy Eating Index; **aMED**, alternate Mediterranean Diet score; **MUFA**, monounsaturated fatty acids; **PUFA**, polyunsaturated fatty acids.

[a]Adjusted for age, gender, smoking, BMI, and energy intake.

Table S4. Association of dietary patterns and components with Parkinson's disease according to gender (N = 181)

		Gender	
		**Men**	**Women**
**Cases/controls**		66/30	32/53
**Dietary pattern**	**Unit**	**OR (95% CI)[a]**	**OR (95% CI)[a]**
HEI	10-point	0.69 (0.42, 1.10)	0.57 (0.31, 1.01)
AHEI	10-point	0.93 (0.53, 1.63)	0.38 (0.20, 0.68)
aMED	1-point	0.85 (0.64, 1.11)	0.73 (0.53, 0.98)
**Nutrient**			
Carbohydrates	10 g/d	1.19 (1.05, 1.37)	1.23 (1.06, 1.46)
Total protein	10 g/d	0.72 (0.53, 0.95)	0.74 (0.47, 1.12)
Total fat	10 g/d	0.88 (0.64, 1.19)	0.70 (0.48, 0.98)
Trans fats	1 g/d	1.86 (1.21, 3.09)	1.38 (0.89, 2.29)
MUFA	10 g/d	0.85 (0.47, 1.55)	0.43 (0.18, 0.89)
PUFA	10 g/d	0.66 (0.27, 1.59)	0.68 (0.27, 1.66)
Total sugars	10 g/d	1.20 (1.05, 1.41)	1.27 (1.09, 1.53)
Added sugars	10 g/d	1.55 (1.24, 2.06)	1.30 (1.09, 1.60)
Fiber	10 g/d	0.67 (0.35, 1.21)	0.31 (0.11, 0.73)
Folate	100 μg/d	0.61 (0.36, 0.96)	0.55 (0.27, 0.98)
Alcohol	10 g/d	0.85 (0.66, 1.08)	0.66 (0.31, 1.17)
Caffeine	100 mg/d	0.84 (0.71, 0.99)	0.51 (0.30, 0.77)
**Food group**			
Vegetables	100 g/d	0.76 (0.45, 1.25)	0.45 (0.17, 0.93)
Fruits	100 g/d	0.83 (0.51, 1.36)	0.97 (0.52, 1.76)
Refined grains	10 g/d	1.05 (0.94, 1.20)	1.03 (0.90, 1.18)
Nuts	100 g/d	0.76 (0.37, 1.56)	0.28 (0.07, 0.83)
Processed meat	10 g/d	0.88 (0.71, 1.09)	1.68 (1.17, 2.58)

Abbreviations: **OR**, odds ratio; **CI**, confidence interval; **HEI**, Healthy Eating Index; **AHEI**, Alternate Healthy Eating Index; **aMED**, alternate Mediterranean Diet score; **MUFA**, monounsaturated fatty acids; **PUFA**, Polyunsaturated fatty acids.

[a]Adjusted for age, smoking, BMI, and energy intake.

Table S5. Associations of dietary patterns and components with Parkinson's disease status using conditional logistic regression with matched pairs (n = 166)

**Dietary Pattern**	**Unit**	**OR (95% CI)**[a]
HEI	10-point	0.69 (0.42, 1.13)
AHEI	10-point	0.65 (0.37, 1.13)
aMED	1-point	0.85 (0.62, 1.17)

Abbreviations: **OR**, odds ratio; **CI**, confidence interval; **HEI**, Healthy Eating Index; **AHEI**, Alternate Healthy Eating Index; **aMED**, alternate Mediterranean Diet score.

[a]Adjusted for age, gender, smoking status, BMI, and energy intake.

Table S6. Association of Parkinson's duration and age at diagnosis with specific nutrient intake (n = 98)

**PD duration**, years	**Unit**	**β (95% CI)**[a]
Carbohydrates	10 g/d	4.15 (-0.11, 8.41)
Trans fats	1 g/d	0.11 (0.02, 0.21)
Total sugars	10 g/d	2.80 (0.29, 5.31)
Added sugars	10 g/d	1.93 (0.10, 3.77)
**Age at diagnosis**, years		
Carbohydrates	10 g/d	-4.08 (-7.99, -0.18)
Trans fats	1 g/d	-0.09 (-0.18, -0.01)
Total sugars	10 g/d	-2.83 (-5.13, -0.54)
Added sugars	10 g/d	-2.32 (-3.98, -0.66)

Abbreviations: **SD**, standard deviation; **PD**, Parkinson's disease; **CI**, confidence interval.
[a]Coefficients are from linear regressions of nutrients on PD duration and age at diagnosis. Adjusted for age and gender.

Table S7. Association of Parkinson's disease clinical features with diet (n = 98)

Clinical feature (present vs. absent)	Unit	β (95% CI)[a]
**Non-motor symptom**[b]		
Trans fats	1 g/d	1.23 (0.31, 2.14)
Refined grains	10 g/d	2.65 (0.24, 5.06)
**Daytime sleepiness**[c]		
Total sugars	10 g/d	2.68 (0.11, 5.24)
Trans fats	1 g/d	1.06 (0.14, 1.98)
**Restless sleep**[d]		
Carbohydrates	10 g/d	6.06 (1.72, 10.39)
Trans fats	1 g/d	1.09 (0.10, 2.07)
Total sugars	10 g/d	3.53 (0.95, 6.10)
**Cognitive impairment**[e]		
Protein	10 g/d	-4.01 (-7.33, -0.68)
Fiber	10 g/d	-1.01 (-1.94, -0.09)
Folate	100 μg/d	-1.30 (-2.52, -0.08)
**Motor symptom**[f]		
AHEI	10-point	-0.36 (-0.73, 0.01)

Abbreviations: **PD**, Parkinson's disease; **UPDRS**, Unified Parkinson's Disease Rating Scale; **NMSS**, Non-Motor Symptom Assessment Scale; **MMSE**, Mini Mental State Exam; **HEI**, Healthy Eating Index; **AHEI**, Alternate Healthy Eating Index; **aMED**, alternate Mediterranean Diet score.

[a]Coeffcients are from linear regressions of dietary patterns and components on clinical features. Adjusted for age and gender.

[b]UPDRS IB non-motor aspects of experiences of daily living items (present: ≥ 9 points; absent: < 9 points).

[c]NMSS daytime sleepiness frequency × severity score (present: > 0 point ; absent: 0 point).

[d]NMSS restless sleep frequency × severity score (present: > 0 point ; absent: 0 point).

[e]MMSE cognitive impairment score (present: < 24 points ; absent: ≥ 24 points).

[f]UPDRS III motor symptom severity and progression (present: ≥ 35 points; absent < 35 points).

REFERENCES

[1] S.M. Krebs-Smith, T.E. Pannucci, A.F. Subar, S.I. Kirkpatrick, J.L. Lerman, J.A. Tooze, M.M. Wilson, J. Reedy, Update of the Healthy Eating Index: HEI-2015, J Acad Nutr Diet 118(9) (2018) 1591-1602.

[2] M.L. McCullough, D. Feskanich, M.J. Stampfer, E.L. Giovannucci, E.B. Rimm, F.B. Hu, D. Spiegelman, D.J. Hunter, G.A. Colditz, W.C. Willett, Diet quality and major chronic disease risk in men and women: moving toward improved dietary guidance, Am J Clin Nutr 76(6) (2002) 1261-71.

[3] T.T. Fung, M.L. McCullough, P.K. Newby, J.E. Manson, J.B. Meigs, N. Rifai, W.C. Willett, F.B. Hu, Diet-quality scores and plasma concentrations of markers of inflammation and endothelial dysfunction, American Journal of Clinical Nutrition 82(1) (2005) 163-173.

[4] A. Trichopoulou, T. Costacou, C. Bamia, D. Trichopoulos, Adherence to a Mediterranean diet and survival in a Greek population, N Engl J Med 348(26) (2003) 2599-608.

[5] A. Trichopoulou, A. Kourisblazos, M.L. Wahlqvist, C. Gnardellis, P. Lagiou, E. Polychronopoulos, T. Vassilakou, L. Lipworth, D. Trichopoulos, Diet and Overall Survival in Elderly People, Bmj-Brit Med J 311(7018) (1995) 1457-1460.

[6] T.T. Fung, F.B. Hu, M.L. McCullough, P.K. Newby, W.C. Willett, M.D. Holmes, Diet quality is associated with the risk of estrogen receptor-negative breast cancer in postmenopausal women., Journal of Nutrition 136(2) (2006) 466-472.
